# Efficacy assessment following shortened venetoclax exposure in AML patients treated with venetoclax plus hypomethylating agents: a real-world, multicentric study

**DOI:** 10.1038/s41408-025-01396-5

**Published:** 2025-10-22

**Authors:** Sonja Wurm, Jennifer M. Moritz, Verena Petzer, Dominik Wolf, Karoline V. Gleixner, Wolfgang R. Sperr, Christina Groiss, Sigrid Machherndl-Spandl, Gregor Eisenwort, Elisabeth Koller, Johannes Schoeche, Clemens Petrasch, Josef Singer, Daniel M. Mayer, Gudrun Pregartner, Annkristin Heine, Albert Wölfler, Heinz Sill, Andreas Reinisch, Armin Zebisch

**Affiliations:** 1 Division of Hematology, Medical University of Graz, Graz, Austria; 2 Department of Internal Medicine V, Haematology and Oncology, Comprehensive Cancer Center Innsbruck (CCCI) and Tyrolean Cancer Research Institute (TKFI), Medical University of Innsbruck (MUI), Innsbruck, Austria; 3 Department of Internal Medicine I, Division of Hematology and Hemostaseology Medical University of Vienna, Vienna, Austria; 4 Division of Hematology with Stem Cell Transplantation, Hemostaseology and Medical Oncology, Department of Internal Medicine I, Ordensklinikum Linz Elisabethinen, Linz, Austria; 5 Medical Faculty, Johannes Kepler University, Linz, Austria; 6 Third Medical Department for Hematology and Oncology, Hanusch Hospital Vienna, Vienna, Austria; 7 Department of Medicine I, Center for Oncology and Hematology, Clinic Ottakring, Vienna, Austria; 8 Karl Landsteiner University of Health Sciences, Krems, Austria; 9 Department of Internal Medicine II, University Hospital Krems, Karl Landsteiner University of Health Sciences, Krems, Austria; 10 Department of Internal Medicine I, Hospital of the Brothers of St. John of God, Graz, Austria; 11 Institute for Medical Informatics, Statistics and Documentation, Medical University of Graz, Graz, Austria; 12 Department of Blood Group Serology and Transfusion Medicine, Medical University of Graz, Graz, Austria; 13 Otto Loewi Research Center for Vascular Biology, Immunology and Inflammation, Division of Pharmacology, Medical University of Graz, Graz, Austria

**Keywords:** Acute myeloid leukaemia, Cancer therapy

Combination therapy of venetoclax (VEN) and hypomethylating agents (HMA) has become the gold-standard first-line treatment for acute myeloid leukemia (AML) patients ineligible for intensive chemotherapy induction [1, 2]. This regimen comprises a standard dose of HMAs (azacitidine [AZA] days 1–7 or decitabine days 1–5 every 28-day cycles) combined with oral, once-daily administration of VEN (continuously in 28-day cycles). While the VIALE-A licensing trial [3] and confirmatory real-world data showed impressive response and survival data for VEN/HMA treatment [4, 5], the myelotoxicity of VEN turned out to be a limiting factor in the routine clinical setting [6, 7].

In a recent article published in this journal, Willekens and colleagues present a retrospective study of patients treated with 7-day AZA plus 7-day VEN in 28-day cycles (7 + 7 regimen) [8]. The authors observed similar response rates compared to standard dose VEN/AZA, suggesting that shorter VEN treatment durations might be sufficient. However, the 7 + 7 regimen is not yet ready for routine clinical use. Even in studies evaluating shortened VEN courses, the optimal duration of VEN administration during each cycle is still a matter of debate [9, 10]. Current routine clinical guidelines do not yet recommend upfront shortened VEN dosing [2, 11]. However, they indirectly support this claim by recommending earlier response assessments. While the VIALE-A licensing trial performed the first efficacy assessment at the end of cycle 1 (±3 days of Cycle 1 Day 28) [3], recent guidelines recommend the first bone marrow (BM) analyses already at day 14–21 [2, 11]. This change to the initial protocol allows for early therapeutic adjustments in neutropenic patients. In case of BM complete remission (mCR, defined as <5% BM blasts and no blasts in the peripheral blood, irrespective of peripheral blood count regeneration), pausing VEN and administering granulocyte-colony stimulating factor (G-CSF) is advised. Ultimately, this procedure might shorten VEN treatment, resulting in a reduced total VEN dose. In contrast, continuation of VEN is recommended in patients with active disease [11].

Within this study, we aimed to further elaborate on earlier response assessment and shortened VEN dosing. Therefore, we retrospectively analyzed a real-world cohort of 184 newly diagnosed AML patients treated with VEN/HMA as first-line therapy to determine whether the number of days of VEN administration before response assessment in treatment cycle 1 affects the frequency of mCR (defined as <5% BM blasts in cytology, flow cytometry and histology). Patients included had VEN-AZA treatment between December 2018 and February 2025 in one of the participating eight Austrian hematology centers. The study was approved by the Medical University of Graz Institutional Review Board (EK-Nr:1337/2024). Statistical analyses were performed using R, version 4.5.0; https://www.R-project.org/ and GraphPad Prism version 10.5.0 (San Diego, CA, USA). All *p*-values are two-sided; the significance level was 0.05. Patient characteristics at the time of diagnosis are listed in Supplementary Table 1; details about VEN/HMA dosage, administration, and the co-administration of CYP3A-influencing drugs are listed in Supplementary Table 2. First, we assessed the duration of VEN therapy during cycle 1, and depending on the BM assessment (BMA), we formed three groups (group 1: BMA after ≤14 days of VEN; group 2: BMA after 15–21 days of VEN; group 3: BMA after 22–28 days of VEN). Overall, mCR was achieved in 113/184 (61%) patients, with comparable mCR frequencies between the groups (Fig. 1A and Supplementary Table 3). These findings were confirmed when CR/CRh was used as the outcome. Yet, as most patients remained neutropenic at the first BMA and therefore did not satisfy CR/CRh criteria, the reliability of these analyses is considerably reduced. (Supplementary Table 3). We then calculated the effect of VEN treatment duration on cycle 1 mCR using logistic regression analysis and did not observe a significant effect (*p* = 0.590; Fig. 1B), which was validated in a multivariable model, including well-recognized AML risk factors (*p* = 0.462, Supplementary Table 4). When examining the other risk factors, we observed significantly lower mCR rates in patients classified within the ELN2024 adverse-risk group in the univariable analysis. However, this association was lost in the multivariable model. It is important to note that these analyses may have been influenced by limited patient numbers in the respective subgroups and by the study protocol itself. The ELN2024 risk model has primarily been validated for overall survival (OS) rather than response after cycle 1 [1, 12]. Ultimately, we corroborated the missing association between shortened VEN duration and mCR achievement by studying VEN treatment duration before BMA in cycle 1 as continuous variable (*p* = 0.721; Fig. 1C). An interesting observation emerged when the total VEN duration in cycle 1 (rather than only the period before the first BMA) was assessed. Patients who had their BMA after ≥22 days of VEN had the longest total VEN exposure in cycle 1, whereas those assessed ≤14 days had the shortest exposure (*p* < 0.001; Supplementary Table 3), suggesting that early response assessment in routine practice resulted in shorter total VEN duration in cycle 1. Indeed, total cycle 1 VEN duration also had no impact on mCR rates when assessed as a continuous variable (Supplementary Fig. 1). We also analyzed the OS and total mCR rates (mCR at any point during therapy) in the three VEN duration groups and found no significant difference (Supplementary Table 3 and Supplementary Fig. 2). However, since the three groups were defined by VEN duration before the first BMA in cycle 1 - and since VEN dosing differed after BMA and in the subsequent cycles - later outcomes like survival or total mCR rates are likely biased. These analyses also highlight the limitations of the retrospective study design. Although the results provide indirect support for the sufficiency of shorter VEN durations, they do not allow for direct conclusions regarding the safety and efficacy of reduced upfront VEN dosing.

**Fig. 1:**
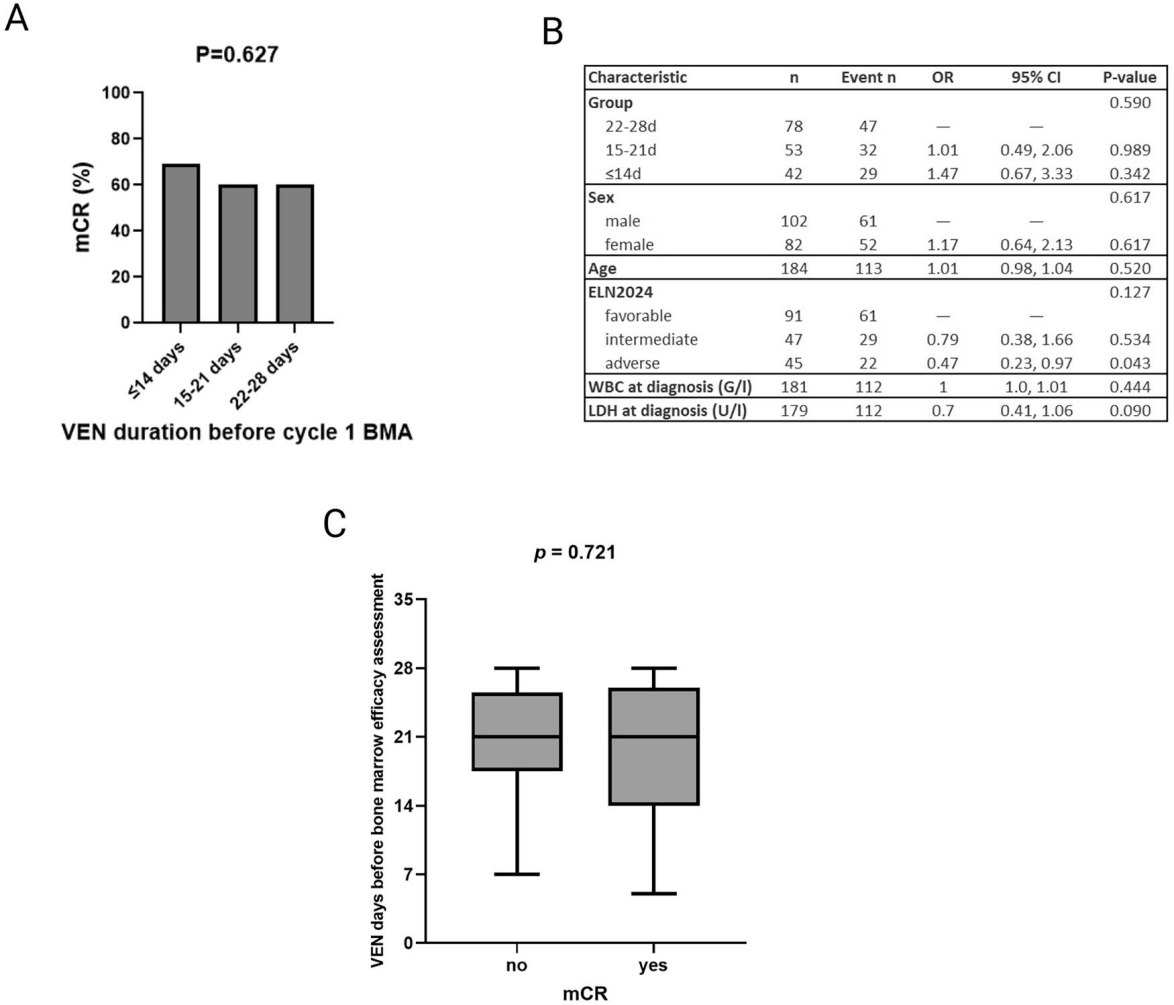
Cycle 1 mCR rates do not differ after shorter and longer VEN administration. **A** Relative mCR frequencies in the 173 patients, where BMA was performed ≤28 days of VEN. Group 1: BMA after ≤14 days of VEN (median 9 days; range 5–14 days); group 2: BMA after 15–21 days of VEN (median 20 days; range 16–21 days); group 3: BMA after 22–28 days of VEN (median 27 days; range 22–28 days). Note that with one exception, all patients in group 1 were evaluated between 7 and 14 days of VEN therapy. Additionally, 11 patients were evaluated after 28 days of VEN and excluded from these analyses. Differences were assessed using Fisher’s exact test. **B** To calculate whether VEN duration before BMA in cycle 1 affects mCR rates, we performed univariable logistic regression analysis, including well-established AML risk factors (WBC white blood cells, LDH lactate dehydrogenase, age age at diagnosis, ELN2024 European LeukemiaNet risk stratification for adults with AML receiving less-intensive therapies). **C** Duration of VEN in days before efficacy assessment as a continuous variable in patients achieving mCR and no mCR. VEN durations between the groups were compared by Mann–Whitney U test. VEN venetoclax, mCR marrow-complete remission, n number, OR Odds Ratio, CI Confidence Interval, d days, G/l Giga per liter.

Another issue of interest with early response assessments arises from the different methods used to assess mCR. While the ELN guidelines recommend assessing AML responses primarily via the aspirate [2], the VIALE-A included BM aspirate and biopsy at the end of cycle 1 [3]. Cycle 2 could only begin once all results were available. Following this protocol raises the problem that although flow cytometry and cytology are usually available quickly, the turnaround time (TAT) for histology is substantially longer. Particularly in neutropenic patients, this discrepancy creates another problem, as G-CSF is recommended in cases where remission has been achieved, while VEN should continue otherwise [13]. Waiting for histology creates a timeframe of uncertainty and might unnecessarily delay G-CSF administration. To elaborate on this topic, we compared the results of BM sampling via flow cytometry, cytology, and histology. The median TAT was 0 days for both flow cytometry and cytology (range 0–4 days for both). However, the histology TAT was considerably longer (median 7 days; range 1–42 days; *p* < 0.001; Fig. 2A). Blast percentages did not differ significantly between these methods, although a trend was observed for lower numbers in flow cytometry (*p* = 0.056; Fig. 2B). All approaches were statistically significantly positively correlated with each other (Fig. 2C). We then repeated the analyses from part 1, asking whether mCR rates between VEN treatment duration groups remained similar when response was assessed by flow cytometry and cytology only. As shown in Supplementary Table 3, this was the case (*p* = 0.850). Importantly, our study also identified occasional cases where the blast percentage in the aspirate was considerably lower than in the histology reports, particularly when flow cytometry alone was used to assess efficacy (Supplementary Fig. 3A). In this respect, we observed that combining flow cytometry with cytology reduced the number of cases that did not match the histology reports (Supplementary Fig. 3B). Moreover, revisiting these patients’ flow cytometry/cytology reports revealed that insufficient quality, such as hemodilution [14, 15], might have biased the lower blast percentages in these cases. Hence, our data highlight that the percentage of blasts in the BM aspirate should be determined based on a combination of cytology and flow cytometry. The results also remind us that information about the sample quality and potential hemodilution should be included in every BM cytology and flow cytometry report. When these measures are implemented, and the BM aspirate is deemed of adequate quality, our data indicate that the results of the BM biopsy should not be awaited.

**Fig. 2:**
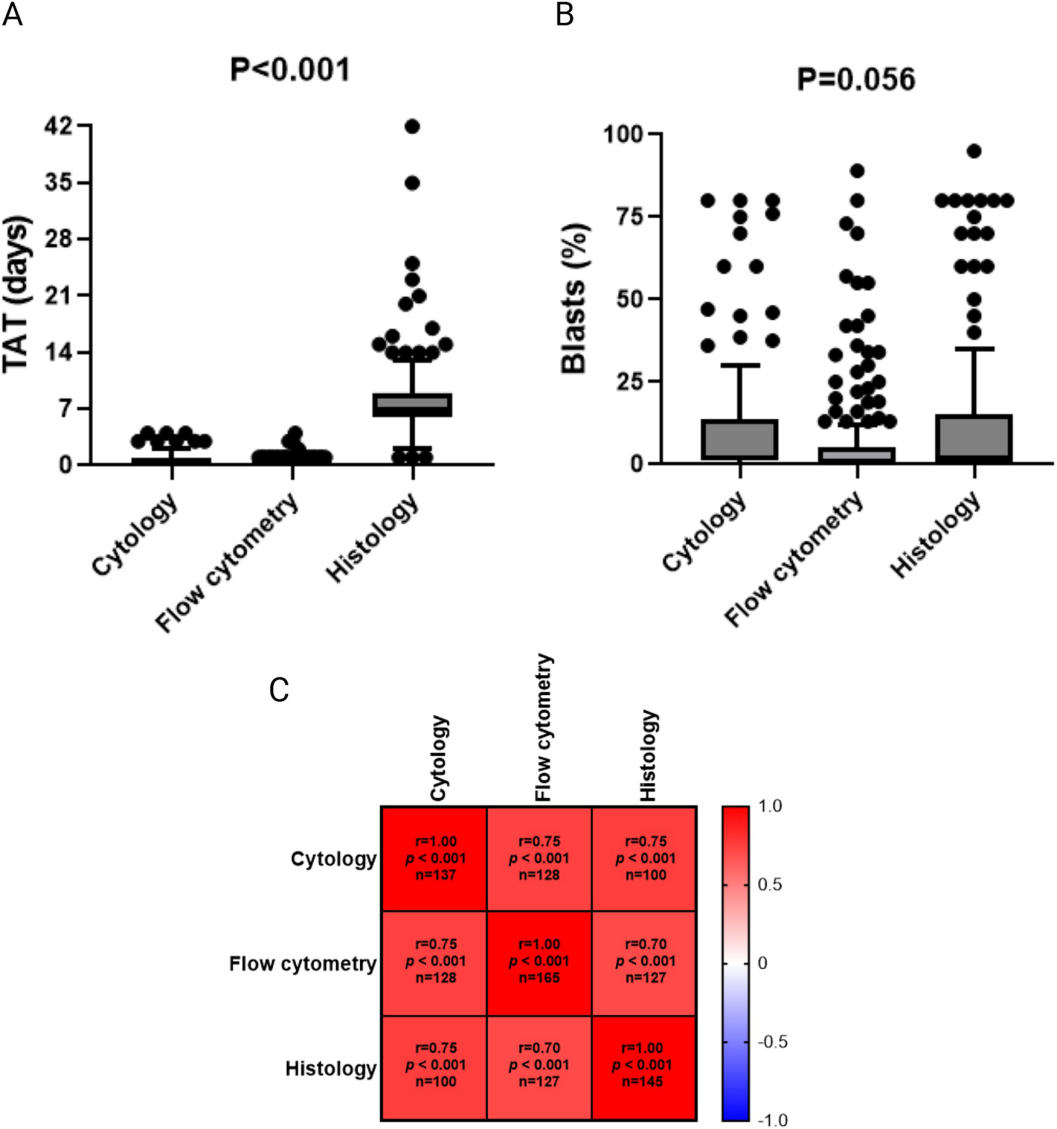
Flow cytometry and cytology can guide therapy after BM assessment in VEN/HMA-treated patients. **A** Turnaround times of histology, flow cytometry, and cytology after BM assessment in cycle 1. Differences were assessed using the Friedman rank sum test. **B** Blast percentages in histology, flow cytometry, and cytology after BM assessment in cycle 1. Overall differences were assessed using the Friedman rank sum test. **C** Heatmap of Spearman correlation analysis between blast percentages in histology, flow cytometry, and cytology after BM assessment in cycle 1. Each field contains the correlation coefficient, the *p*-value, and the number of patients with the respective assessments. TAT turnaround time.

In conclusion, we retrospectively analyze first-line VEN/HMA treatment in 184 AML patients and demonstrate that the first response assessment can be safely performed after 7 to 14 days of VEN administration. This procedure enables earlier VEN pausing in case of neutropenia. The data also support the claim that shorter VEN treatment durations may be sufficient for AML treatment regimens. Secondly, we demonstrate that BM analysis performed via flow cytometry, cytology, and histology yields comparable results when the quality of the aspirates is adequate. Consequently, we propose that the results of the BM biopsy should not be awaited in VEN/HMA-treated AML patients and that the decision to pause or continue VEN can be safely made based on flow cytometry and cytology results with adequate quality alone.

## Supplementary information

Supplementary data
