# Supplementary material for: Efficacy assessment following shortened venetoclax exposure in AML patients treated with venetoclax plus hypomethylating agents: a real-world, multicentric study
Source: Blood Cancer J. 2025 Oct 22;15(1):175. doi: 10.1038/s41408-025-01396-5 (PMC12546751; doi:10.1038/s41408-025-01396-5)

**Supplementary data:**

**Efficacy assessment following shortened venetoclax exposure in AML patients treated with venetoclax plus hypomethylating agents: a real-world, multicentric study**

Wurm S et al.

**Supplementary Table 1: Description of the cohort**

Characterization of the 184 AML patients studied. Categorical parameters are presented as n (%) and continuous variables as median (range). LDH, lactate dehydrogenase; WBC, white blood cell count; ELN, European LeukemiaNet.

| **Age at diagnosis (years)** | 73 (24-94) |
| --- | --- |
| **AML subtype** |  |
| de novo | 115 (62%) |
| Secondary | 69 (38%) |
| **Sex** |  |
| Male | 102 (55%) |
| Female | 82 (45%) |
| **LDH at diagnosis (U/L)** | 321 (110-6701) |
| **WBC at diagnosis (10^9^/L)** | 4 (0-181) |
| **ELN2024 risk group** |  |
| Favorable | 90 (49%) |
| Intermediate | 47 (26%) |
| Adverse | 46 (25%) |
| data not available | 1 |

**Supplementary Table 2: Details about VEN/HMA administration before response assessment in Cycle 1**

Categorical parameters are presented as n (%) and continuous variables as median (range). VEN, venetoclax; BMA, bone marrow assessment, CYP3A, Cytochrome P450, family 3, subfamily A; d, days.*VEN was dosed according to the label with dose reductions in case of co-medication with CYP3A inhibitors. §Decitabine and Azacitidine were dosed according to the label.

| **Days of VEN before BMA (d)*** | 21 (5-28) |
| --- | --- |
| **Co-medication with strong CYP3A inhibitor** |  |
| Yes | 88 (50%) |
| No | 89 (50%) |
| data not available | 7 |
| **Co-medication with medium CYP3A inhibitor** |  |
| Yes | 2 (1%) |
| No | 156 (99%) |
| data not available | 26 |
| **Hypomethylating agent used** |  |
| Azacitidine | 182 (99%) |
| Decitabine | 2 (1%) |
| **Days of Decitabine before BMA (d)§** | 5 (5-5) |
| **Days of Azacitidine before BMA (d)§** | 7 (5-7) |

**Supplementary Table 3: Description of the three VEN duration groups**

Categorical parameters are presented as n (%) and compared with Fisher’s exact test. Continuous variables are presented as median (range) and compared with the Mann Whitney U test. VEN, venetoclax; BMA, bone marrow assessment; AML, acute myeloid leukemia; LDH, lactate dehydrogenase; WBC, white blood cell count; ELN, European LeukemiaNet; CR, complete remission; CRm, bone marrow CR; CRh, CR with partial hematological recovery

|  | **Group 1**  (VEN duration ≤14 days before cycle 1 BMA)  **(n=42)** | **Group 2**  (VEN duration 15-21 days before cycle 1 BMA)  **(n=53)** | **Group 3**  (VEN duration 22-28 days before cycle 1 BMA)  **(n=78)** | ***p* - value** |
| --- | --- | --- | --- | --- |
| **Age at diagnosis (years)** | 71 (33-86) | 74 (24-84) | 75 (27-94) | 0.132 |
| **AML subtype** |  |  |  | 0.793 |
| De novo | 25 (60%) | 35 (66%) | 48 (62%) |  |
| Secondary | 17 (40%) | 18 (34%) | 30 (38%) |  |
| **Sex** |  |  |  | >0.999 |
| Male | 23 (55%) | 29 (55%) | 42 (54%) |  |
| Female | 19 (45%) | 24 (45%) | 36 (46%) |  |
| **LDH at diagnosis (U/L)** | 397 (126-6701) | 272 (129-1399) | 321 (110-3057) | 0.080 |
| **WBC at diagnosis (10^9^/L)** | 10.2 (0.6-169) | 3.5 (0.5-124.8) | 3.3 (0.3-181.2) | 0.021 |
| **ELN2024 risk group** |  |  |  | 0.089 |
| Favorable | 18 (44%) | 24 (45%) | 47 (60%) |  |
| Intermediate | 15 (37%) | 11 (21%) | 16 (21%) |  |
| Adverse | 8 (19%) | 18 (34%) | 15 (19%) |  |
| Data not available | 1 | 0 | 0 |  |
| **CRm** | 29/42 (69%) | 32/53 (60%) | 47/78 (60%) | 0.627 |
| **CR/CRh** | 6/41 (15%) | 1/53 (2%) | 8/77 (10%) | 0.060 |
| **CRm flow/cyto** (assessed by flow cytometry/cytology only) | 29/41 (71%) | 33/50 (66%) | 49/75 (65%) | 0.850 |
| **CRm final** (at any point during VEN/HMA treatment) | 31/41 (76%) | 37/53 (70%) | 56/78 (72%) | 0.862 |
| **Diagnosis time** |  |  |  | <0.001 |
| 2018-2022 | 6/42 (14%) | 14/53 (26%) | 49/78 (63%) |  |
| 2023-2025 | 36/42 (86%) | 39/53 (74%) | 29/78 (37%) |  |
| **(Cyto)Genetics** |  |  |  |  |
| *IDH1/2* | 9/42 (21%) | 13/52 (25%) | 16/78 (21%) | 0.842 |
| *NRAS/KRAS* | 11/41 (27%) | 6/52 (12%) | 8/78 (10%) | 0.055 |
| *FLT3-ITD* | 9/42 (21%) | 6/53 (11%) | 6/78 (8%) | 0.102 |
| *TP53* | 8/41 (20%) | 10/53 (19%) | 14/78 (18%) | 0.968 |
| *DDX41* | 1/6 (17%) | 0/34 (0%) | 2/42 (5%) | 0.109 |
| CBF – t(8;21), inv(16) | 1/34 (3%) | 1/48 (2%) | 3/72 (4%) | 0.855 |
| Complex CG | 7/40 (18%) | 11/50 (22%) | 10/73 (14%) | 0.480 |
| **VEN duration before BMA cycle 1 (days)** | 9 (5-14) | 20 (16-21) | 27 (22-28) | <0.001 |
| **Total VEN duration cycle 1 (days)** | 9 (5-27) | 21 (17-28) | 28 (22-28) | <0.001 |

**Supplementary Table 4: Cycle 1 mCR rates do not differ after shorter and longer VEN administration.**

To calculate whether VEN duration before BMA in cycle 1 affects mCR rates, we performed multivariable logistic regression analysis, including well-established AML risk factors (WBC, white blood cells; LDH, lactate dehydrogenase; Age, age at diagnosis; ELN2024, European LeukemiaNet risk stratification for adults with AML receiving less-intensive therapies) in the latter. n, number; OR, Odds Ratio; CI, Confidence Interval; d, days; G/l, Giga per liter

**Supplementary Figure 1: Total duration of VEN in C1 has no effects on mCR rates.**

Duration of VEN during cycle 1 in days as a continuous variable in patients achieving mCR and no mCR. VEN durations between the groups were compared by Mann-Whitney-U test. VEN, venetoclax; mCR, marrow-complete remission

**Supplementary Figure 2: Overall survival does not differ between the three groups of VEN duration before BMA in cycle 1.**

Kaplan-Meier analysis for: A, Overall survival (OS) in the total cohort studied. B, OS in the three groups of VEN duration before BMA in cycle 1 (group 1: BMA after ≤14 days of VEN; group 2: BMA after 15-21 days of VEN; group 3: BMA after 22-28 days of VEN). Differences between groups were assessed using the log-rank test.


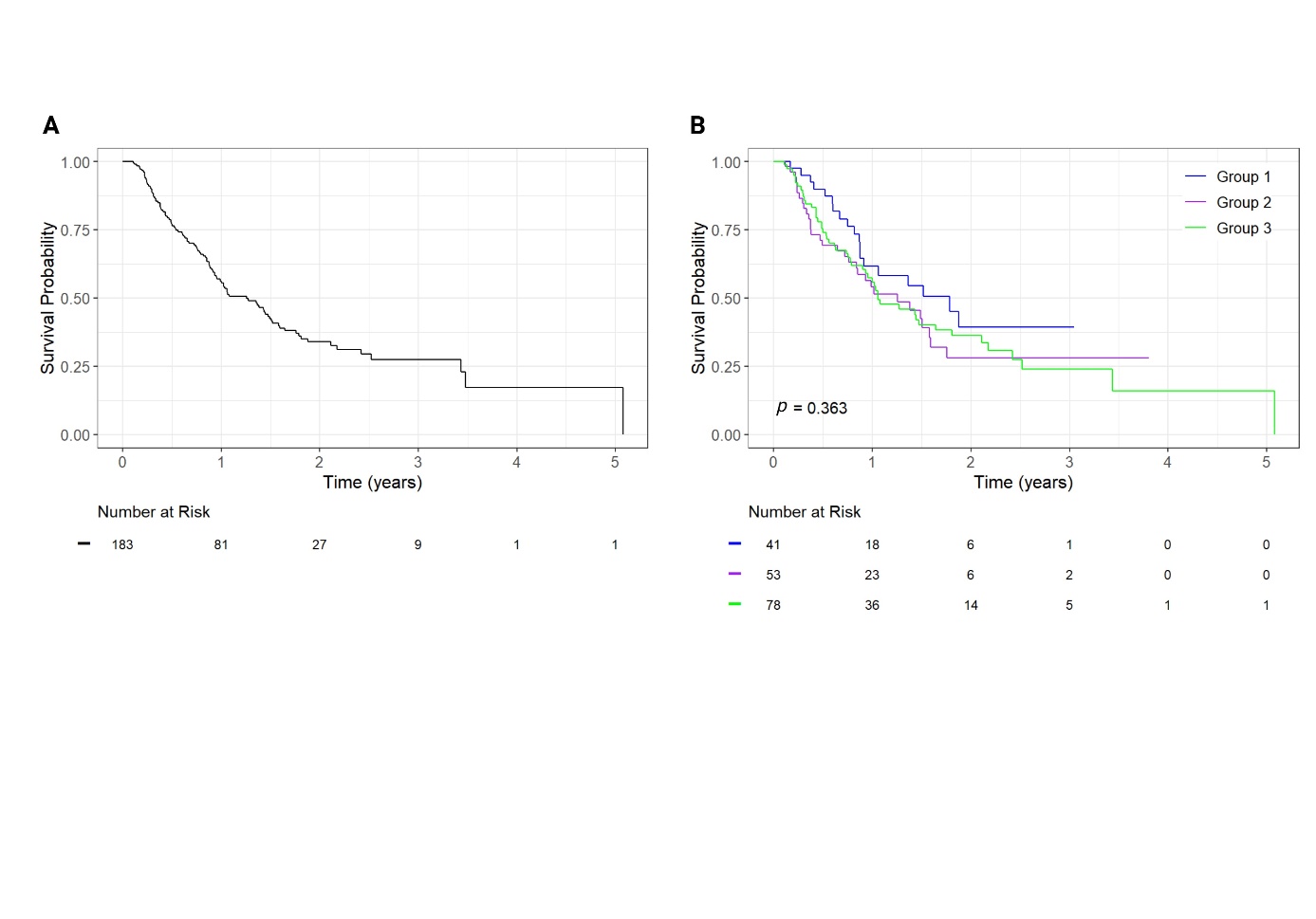


**Supplementary Figure 3: Accuracy of flow cytometry/cytology in predicting mCR.**

**A,** Flow chart showing the accuracy of flow cytometry in the prediction of CR. 80/93 (86%) of patients classified as mCR in flow cytometry were validated by histology, whereas 13/93 (14%) were re-classified as “no mCR”. Insufficient sample quality (hemodilution) was reported in most of these cases, some reports did not report sample quality at all. **B,** Flow chart showing the accuracy of combining cytology and flow cytometry in the prediction of CR. Together 85/91 (93%) of patients classified as mCR in cytology/flow cytometry were validated by histology, whereas 6/91 (7%) were re-classified as “no mCR”. Insufficient sample quality (hemodilution) was reported in most of these cases, some reports did not report sample quality at all. TAT, turnaround time; BMA, bone marrow aspirate; mCR, marrow-complete remission


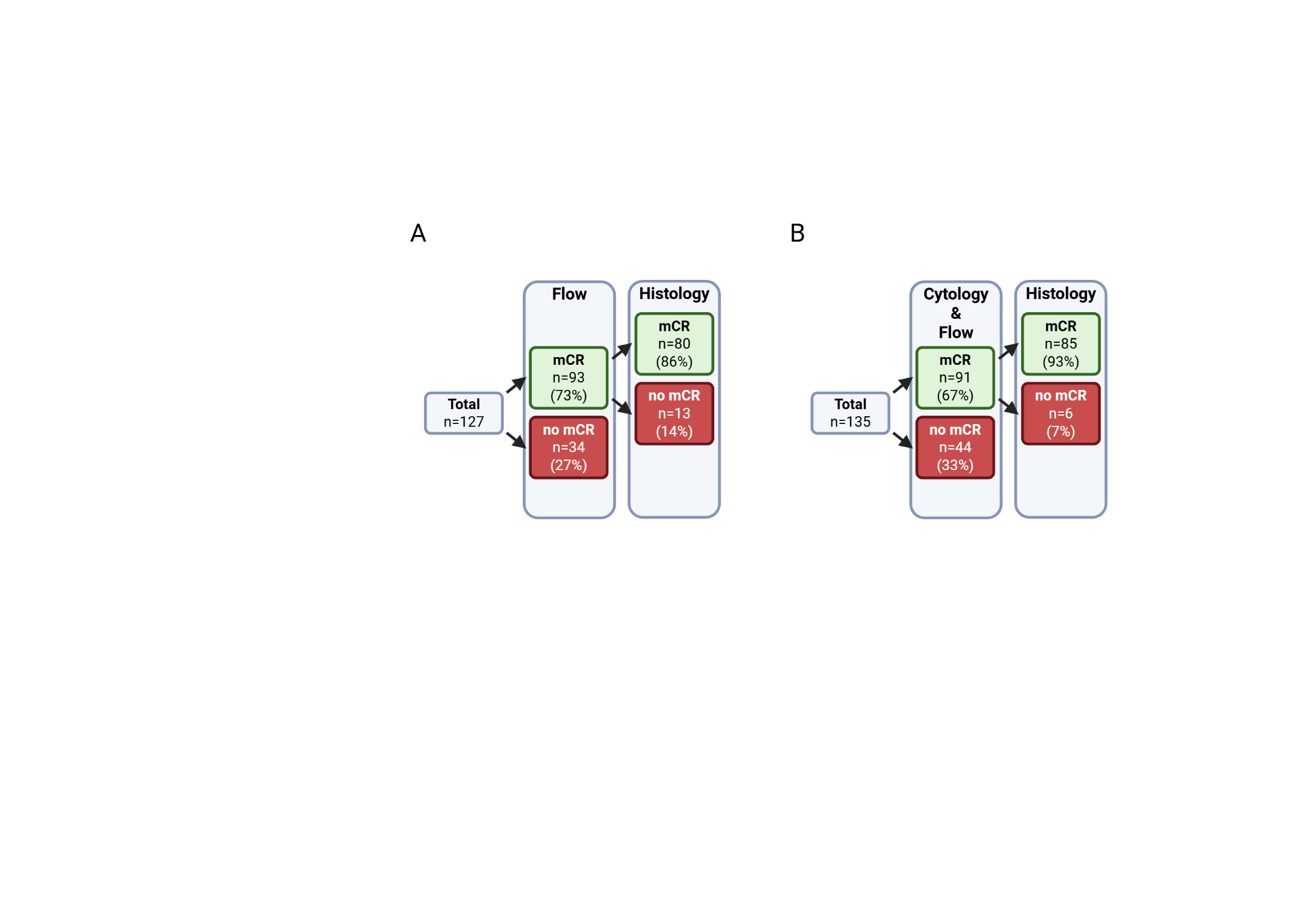

Supplement: Supplementary file 1 — Supplementary data [file 41408_2025_1396_MOESM1_ESM.docx]
